# Effect of Ghrelin on Hepatic IGF-Binding Protein-1 Production

**DOI:** 10.1155/2013/751401

**Published:** 2013-01-17

**Authors:** Moira S. Lewitt

**Affiliations:** School of Health, Nursing and Midwifery, University of the West of Scotland, Paisley Campus, Paisley PA1 2BE, UK

## Abstract

Ghrelin plays key roles in energy homeostasis by central and peripheral actions that include effects on insulin signalling pathways in liver. Insulin is an important inhibitor of production by hepatocytes of insulin-like growth factor-binding protein-1 (IGFBP-1) which has an endocrine role to inhibit IGF availability. The effects of ghrelin, insulin, an AMPK activator, and an AMPK inhibitor on IGFBP-1 secretion were studied in H4-II-E rat liver cells. Ghrelin (100 nM) blocked the inhibitory effect of a maximally effective concentration of insulin (10 ng/mL) on IGFBP-1 secretion during a 5 h incubation period (*P* < 0.001) in the absence and presence of an AMPK inhibitor. Ghrelin, alone, had no effect on IGFBP-1 production, but enhanced secretion independently of insulin under conditions of AMPK activation (*P* < 0.001). In conclusion, IGFBP-1 is identified as a novel target of ghrelin action in liver that may contribute to its metabolic effects in obesity.

## 1. Introduction

Insulin-like growth factor-binding protein-1 (IGFBP-1) is a member of a family of six IGFBPs, which have effects on cell metabolism, motility, growth, and survival via IGF-dependent and -independent mechanisms [1]. Liver is the most important source of IGFBP-1 in the human circulation and hepatic *Igfbp1* transcription is inhibited by the action of portal insulin [2]. As a consequence, there is an inverse relationship between circulating insulin and IGFBP-1 concentrations [2, 3]. This relationship is preserved in obesity [4]. In adults with type 2 diabetes, however, there is an upward shift in the regression line so that IGFBP-1 levels are higher than expected for a given insulin concentration [3]. This is consistent with a decrease in hepatic insulin sensitivity or a reduction in hepatic insulin extraction [3], or an increase in factors that stimulate IGFBP-1 directly, including pro-inflammatory cytokines [5].

Although IGFBP-1 levels in simple obesity are appropriately low for the prevailing hyperinsulinemia, in patients with obesity due to the Prader-Willi syndrome, IGFBP-1 concentrations are not suppressed [6]. Interestingly, circulating levels of the gut peptide ghrelin are also elevated in this syndrome [7, 8], whilst they are low in simple obesity [9, 10]. Like IGFBP-1 [11], ghrelin has a glucose counter-regulatory role [12]. Ghrelin is essential for blood glucose control in starvation [13, 14]. These metabolic effects are mediated in part by a central stimulatory effect of ghrelin on appetite and GH release and in part by peripheral actions on insulin secretion and insulin sensitivity, and hepatic glucose production [12, 15].

We have previously used the insulin-sensitive rat hepatoma cell line H4-II-E to explore the factors regulating IGFBP-1 production. Stimulating AMP-activated protein kinase (AMPK) increases hepatic IGFBP-1 secretion and attenuates the inhibitory effect of insulin [16]. Acylated ghrelin is reported to have a direct effect on intracellular insulin receptor signaling in this cell line [17]. The aim of this study therefore was to determine the short term effect of acylated ghrelin on IGFBP-1 secretion by H4-II-E cells.

## 2. Methods

### 2.1. Reagents

Acylated ghrelin and 5-aminoimidazole-4-carboxamide-ribonucleoside (AICAR) were purchased from Sigma-Aldrich (Sweden). The AMPK inhibitor, 6-[4-(2-Piperidin-1-yl-ethoxy)-phenyl)]-3-pyridin-4-yl-pyrrazolo[1,5-a]-pyrimidine (compound C), was from Calbiochem (EMD Biosciences, San Diego, CA), and recombinant human insulin (Actrapid) from Novo-Nordisk (Sweden).

### 2.2. Cell Culture

H4-II-E cells, obtained from ATCC (Manassas, VA), were maintained in DMEM, 10% fetal bovine serum in 5% CO_2_ in a humidified incubator, and subcultured at a 1 : 5 split ratio twice a week. Cells were plated for experiments in 96-place multiwells (Costar, Corning, NY) and made serum-free for 24 h, before experiments were performed on confluent cells in 200 *μ*L serum-free medium. Under these conditions 4–7 ng/well IGFBP-1 were secreted during a 5 h incubation period. Ghrelin, AICAR, and compound C were stored as concentrated stocks in DMSO. The highest final concentration of DMSO in the cell experiments was 0.1% which had no effect on IGFBP-1 secretion.

### 2.3. IGFBP-1 Immunoassay

Rat IGFBP-1 in conditioned medium was measured by immunoassay, as previously described [16]. In brief, rat IGFBP-1 was used as the fixed antigen applied to Immulon 2 plates (Dynex Technologies, Chantilly, VA) competing for rabbit anti-rat IGFBP-1 antibody B4, 1 : 10,000 with rat IGFBP-1 standard or sample containing an unknown concentration of IGFBP-1. After incubation in 100 *μ*L assay buffer (0.1 M sodium phosphate buffer, 0.1 M sodium chloride, pH 7.4, containing 0.1% Tween (USB, Cleveland, OH)) for 16 h at 4°C the plates were washed twice with assay buffer and bound antibody detected with goat anti-rabbit *γ*-globulin biotin conjugate (Sigma-Aldrich) followed by neutravidin-horseradish peroxidase conjugate (Pierce, Rockford, IL). After incubation with tetramethylbenzidine free base (Sigma-Aldrich), the reaction was stopped with 2 M sulfuric acid and read spectrophotometrically at 450 nm. The effective assay range was 0.05 to 10 ng/assay well. Over 10 assays, nonspecific binding was 3.9 ± 0.4% and half maximal displacement of specifically bound antibody at 0.87 ± 0.04 ng of rat IGFBP-1 standard. At 0.9 ng/well the between- and within-assay imprecision was 15% and 4.8%, respectively (*n* = 10). Samples from one experiment were assayed together using 50 *μ*L of medium.

### 2.4. Statistics

Each experiment presented in this paper was repeated on 3–5 occasions with 3–6 replicates within each experiment for each condition. Data are expressed as a percentage of control, which was set to 100%. Treatment effects were calculated as the percentage of control within each separate experiment. The values shown are the mean ± SEM for the pooled experiments. Statistical significance, taken as *P* < 0.01, was determined by 2-way analysis of variance followed by a multiple comparisons procedure (Student-Newman-Keuls method).

## 3. Results

**Figure 1:**
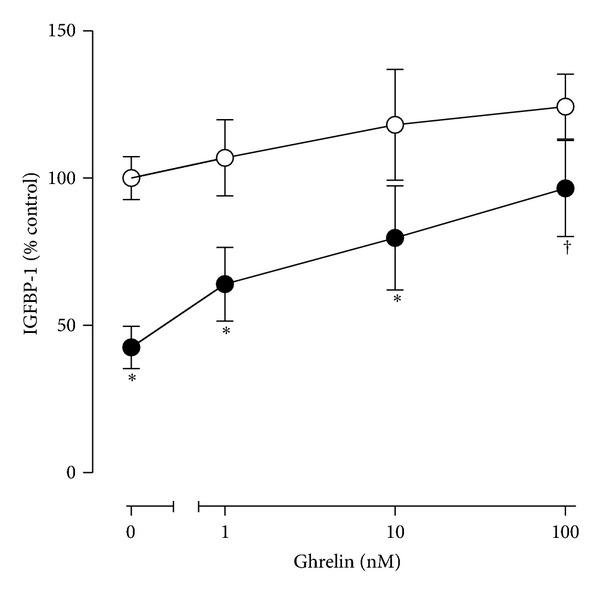
The inhibitory effect of insulin on IGFBP-1 secretion by H4-II-E cells is attenuated in the presence of ghrelin. Confluent cells were exposed to increasing concentrations of ghrelin in the absence (open circles) and presence (closed circles) of insulin, for a 5 h incubation period under serum-free conditions. IGFBP-1 concentrations, measured by specific immunoassay, pooled from three experiments each containing 3-4 replicates per condition, are expressed as a percentage of the secretion by control wells. *P < 0.001 (effect of insulin at a given concentration of ghrelin); ^†^ *P* < 0.001 (effect of ghrelin in the presence of insulin).

The effect of acylated ghrelin on IGFBP-1 secretion was studied in H4-II-E rat liver cells, in the presence and absence of a maximally effective inhibitory concentration of insulin (10 ng/mL). Insulin inhibited IGFBP-1 secretion by H4IIE cells by 60% during a 5 h incubation (*P* < 0.001, Figure 1). This was partially attenuated by increasing concentrations of ghrelin, so that concentrations of IGFBP-1 were higher in the presence of 100 nM ghrelin and insulin, compared to insulin alone (*P* < 0.001). In the absence of insulin, ghrelin had no significant effect on IGFBP-1 production.

**Figure 2:**
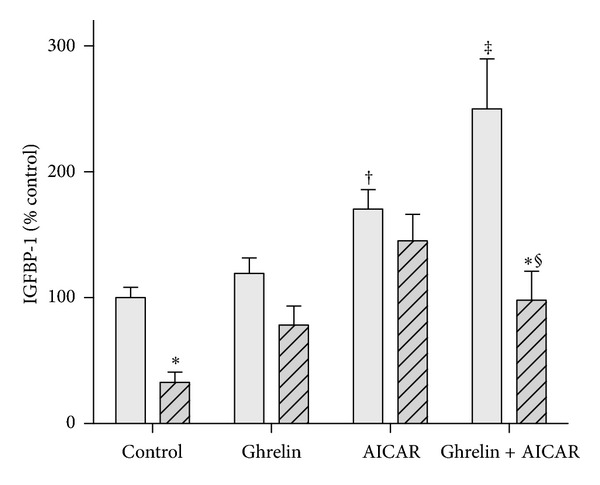
Ghrelin enhances the stimulatory effect of AICAR on IGFBP-1 secretion by H4-II-E cells. Confluent cells were exposed to ghrelin (100 nM) and AICAR (200 *μ*M) in the absence and presence (hatched bars) of 10 ng/mL insulin for a 5 h incubation period under serum-free conditions. IGFBP-1 concentrations, measured by specific immunoassay, pooled from three experiments each containing 4–6 replicates per condition, are expressed as a percentage of the secretion by control wells. *P < 0.001 (effect of insulin compared to condition in the absence of insulin); ^†^ *P* < 0.001 (effect of AICAR compared to control wells); ^‡^ *P* < 0.001 (compared to AICAR alone); ^§^ *P* < 0.001 (compared to insulin alone).

Compound C (0.1–100 *μ*M), a specific inhibitor of AMPK, failed to abolish ghrelin's effect on IGFBP-1 in the presence of insulin (data not shown). Shown in Figure 2, a maximally effective concentration of AICAR (200 *μ*M), which activates AMPK, stimulated IGFBP-1 production by 75% and completely abolished the inhibitory effect of insulin. In the presence of 100 nM ghrelin, the action of AICAR was enhanced (*P* < 0.001, compared to AICAR alone). When cells were exposed to ghrelin and AICAR together, insulin inhibited IGFBP-1 production approx 60% (*P* < 0.001, compared to ghrelin and AICAR).

## 4. Discussion

Ghrelin and its receptors have a widespread distribution and multiple peripheral actions. These include effects on liver, where ghrelin is reported to have both insulin-like [17, 18] and insulin-opposing actions [17]. It has been shown that ghrelin stimulates proliferation of HepG2 cells through upregulation of the IRS-1-GRB2-MAPK pathway [17]. However it inhibits the IRS-1-PI3 K-Akt pathway, and this is likely to be how it opposes the inhibitory effect of insulin on PEPCK expression [17] and stimulates hepatic glucose production [17, 18]. It therefore seems likely that this is also the mechanism whereby it blocks the inhibitory effect of insulin on IGFBP-1 synthesis. Observations of ghrelin's metabolic actions *in vitro* are supported by the responses observed after ghrelin administration *in vivo*. Ghrelin-induced hyperglycaemia in rats is associated with increased hepatic glucose-6-phosphatase gene expression [19] and with suppressed Akt phosphorylation, reduced phosphorylation of glycogen synthase kinase 3, and an increase in *Ppargc1a* gene expression [20]. In mice, administration of ghrelin prevents suppression of endogenous glucose production by insulin [21].

Since AMPK activity inhibits PEPCK expression, it has been proposed that AMPK inhibition might be a mechanism by which ghrelin stimulates gluconeogenesis [22]. In the studies presented here, the pattern of responses to ghrelin was similar to those previously observed for PEPCK [17]; however the failure of compound C to abolish the effect of ghrelin on IGFBP-1 in the presence of insulin suggests that the pathways involved in this action are independent of AMPK. I have previously shown that AICAR, an activator of AMPK, in contrast to its inhibitory effect on PEPCK expression, stimulates hepatic IGFBP-1 secretion [16]. I now demonstrate that there is synergism between AICAR and ghrelin in stimulating hepatic IGFBP-1, suggesting that ghrelin has an action downstream of AMPK. Thus, there appear to be two pathways regulating IGFBP-1 production that involve AMPK, one of which is inhibited by insulin. In adults IGFBP-1 is primarily produced in liver, but is also present in deciduum, where it has a paracrine role [23]. In human endometrial stromal cells ghrelin enhances decidualisation, which is reflected in an increase in local IGFBP-1 production [24]. Direct effects of ghrelin on decidual IGFBP-1 transcription are yet to be explored.

IGFBP-1 and ghrelin appear to have complementary physiological roles. They have similar patterns of nutritional regulation *in vivo* [4, 6, 7, 9]. Like IGFBP-1 [3, 25, 26], a low fasting ghrelin level is a marker of the metabolic syndrome [27–30]. In Prader-Willi syndrome ghrelin is elevated to levels not explained by relative insulin sensitivity and lower insulin levels [31]. It may be that the relatively high IGFBP-1 levels in this syndrome have a similar underlying mechanism. IGFBP-1 and ghrelin both increase blood glucose when administered in pharmacological concentrations, albeit by different mechanisms. The effect of IGFBP-1 on blood glucose is due to it regulation of IGF availability [32], whereas ghrelin has centrally initiated endocrine effects but also has direct effects peripherally, reducing insulin secretion [12, 15], shifting fuel utilisation from lipid to carbohydrate thereby inducing adiposity [33] and increasing gluconeogenesis [17, 18]. Antagonists of ghrelin-activated pathways are regarding as promising in the future treatment of obesity and diabetes [15, 34].

## 5. Conclusion

These experiments add to knowledge of the complex pathways regulating IGFBP-1. Ghrelin blocks the inhibitory effect of insulin on IGFBP-1 secretion from liver cells and, in the presence of AMPK activation, stimulates IGFBP-1 production. Thus IGFBP-1 is identified as a novel target of ghrelin action in liver that may contribute to the spectrum of metabolic actions of ghrelin in obesity.

## References

[1] Firth SM, Baxter RC (2002). Cellular actions of the insulin-like growth factor binding proteins. *Endocrine Reviews*.

[2] Yki-Järvinen H, Makimattila S, Utriainen T, Rutanen EM (1995). Portal insulin concentrations rather than insulin sensitivity regulate serum sex hormone-binding globulin and insulin-like growth factor binding protein 1 *in vivo*. *Journal of Clinical Endocrinology and Metabolism*.

[3] Lewitt MS, Hilding A, Östenson CG, Efendic S, Brismar K, Hall K (2008). Insulin-like growth factor-binding protein-1 in the prediction and development of type 2 diabetes in middle-aged Swedish men. *Diabetologia*.

[4] Conover CA, Lee PDK, Kanaley JA, Clarkson JT, Jensen MD (1992). Insulin regulation of insulin-like growth factor binding protein-1 in obese and nonobese humans. *Journal of Clinical Endocrinology and Metabolism*.

[5] Lang CH, Nystrom GJ, Frost RA (1999). Regulation of IGF binding protein-1 in Hep G2 cells by cytokines and reactive oxygen species. *American Journal of Physiology*.

[6] Höybye C, Frystyk J, Thorén M (2003). The growth hormone-insulin-like growth factor axis in adult patients with Prader Willi syndrome. *Growth Hormone and IGF Research*.

[7] Cummings DE, Clement K, Purnell JQ (2002). Elevated plasma ghrelin levels in Prader-Willi syndrome. *Nature Medicine*.

[8] Delparigi A, Tschöp M, Heiman ML (2002). High circulating ghrelin: a potential cause for hyperphagia and obesity in Prader-Willi syndrome. *Journal of Clinical Endocrinology and Metabolism*.

[9] Tschöp M, Weyer C, Tataranni PA, Devanarayan V, Ravussin E, Heiman ML (2001). Circulating ghrelin levels are decreased in human obesity. *Diabetes*.

[10] Shiiya T, Nakazato M, Mizuta M (2002). Plasma ghrelin levels in lean and obese humans and the effect of glucose on ghrelin secretion. *Journal of Clinical Endocrinology and Metabolism*.

[11] Lewitt MS, Baxter RC (1991). Insulin-like growth factor-binding protein-1: a role in glucose counterregulation?. *Molecular and Cellular Endocrinology*.

[12] Delhanty PJ, van der Lely AJ (2011). Ghrelin and glucose homeostasis. *Peptides*.

[13] Zhao TJ, Liang G, Li RL (2010). Ghrelin O-acyltransferase (GOAT) is essential for growth hormone-mediated survival of calorie-restricted mice. *Proceedings of the National Academy of Sciences of the United States of America*.

[14] Sun Y, Butte NF, Garcia JM, Smith RG (2008). Characterization of adult ghrelin and ghrelin receptor knockout mice under positive and negative energy balance. *Endocrinology*.

[15] Verhulst PJ, Depoortere I (2012). Ghrelin's second life: from appetite stimulator to glucose regulator. *World Journal of Gastroenterology*.

[16] Lewitt MS (2001). Stimulation of IGF-binding protein-1 secretion by AMP-activated protein kinase. *Biochemical and Biophysical Research Communications*.

[17] Murata M, Okimura Y, Iida K (2002). Ghrelin modulates the downstream molecules of insulin signaling in hepatoma cells. *The Journal of Biological Chemistry*.

[18] Gauna C, Delhanty PJD, Hofland LJ (2005). Ghrelin stimulates, whereas des-octanoyl ghrelin inhibits, glucose output by primary hepatocytes. *Journal of Clinical Endocrinology and Metabolism*.

[19] Barazzoni R, Bosutti A, Stebel M (2005). Ghrelin regulates mitochondrial-lipid metabolism gene expression and tissue fat distribution in liver and skeletal muscle. *American Journal of Physiology*.

[20] Barazzoni R, Zanetti M, Cattin MR (2007). Ghrelin enhances *in vivo* skeletal muscle but not liver AKT signaling in rats. *Obesity*.

[21] Heijboer AC, van den Hoek AM, Parlevliet ET (2006). Ghrelin differentially affects hepatic and peripheral insulin sensitivity in mice. *Diabetologia*.

[22] Xue B, Kahn BB (2006). AMPK integrates nutrient and hormonal signals to regulate food intake and energy balance through effects in the hypothalamus and peripheral tissues. *Journal of Physiology*.

[23] Westwood M (1999). Role of insulin-like growth factor binding protein 1 in human pregnancy. *Reviews of Reproduction*.

[24] Tawadros N, Salamonsen LA, Dimitriadis E, Chen C (2007). Facilitation of decidualization by locally produced ghrelin in the human endometrium. *Molecular Human Reproduction*.

[25] Lewitt MS, Hilding A, Brismar K, Efendic S, Östenson CG, Hall K (2010). IGF-binding protein 1 and abdominal obesity in the development of type 2 diabetes in women. *European Journal of Endocrinology*.

[26] Rajpathak SN, He M, Sun Q (2012). Insulin-like growth factor axis and risk of type 2 diabetes in women. *Diabetes*.

[27] Ukkola O, Pöykkö SM, Kesäniemi YA (2006). Low plasma ghrelin concentration is an indicator of the metabolic syndrome. *Annals of Medicine*.

[28] Ikezaki A, Hosoda H, Ito K (2002). Fasting plasma ghrelin levels are negatively correlated with insulin resistance and PAI-1, but not with leptin, in obese children and adolescents. *Diabetes*.

[29] Katsuki A, Urakawa H, Gabazza EC (2004). Circulating levels of active ghrelin is associated with abdominal adiposity, hyperinsulinemia and insulin resistance in patients with type 2 diabetes mellitus. *European Journal of Endocrinology*.

[30] Pöykkö SM, Kellokoski E, Hörkkö S, Kauma H, Kesäniemi YA, Ukkola O (2003). Low plasma ghrelin is associated with insulin resistance, hypertension, and the prevalence of type 2 diabetes. *Diabetes*.

[31] Goldstone AP, Patterson M, Kalingag N (2005). Fasting and postprandial hyperghrelinemia in Prader-Willi syndrome is partially explained by hypoinsulinemia, and is not due to peptide YY 3-36 deficiency or seen in hypothalamic obesity due to craniopharyngioma. *Journal of Clinical Endocrinology and Metabolism*.

[32] Murphy LJ (2003). The role of the insulin-like growth factors and their binding proteins in glucose homeostasis. *Experimental Diabesity Research*.

[33] Tschop M, Smiley DL, Heiman ML (2000). Ghrelin induces adiposity in rodents. *Nature*.

[34] Patterson M, Bloom SR, Gardiner JV (2011). Ghrelin and appetite control in humans—potential application in the treatment of obesity. *Peptides*.

